# Small Endogenous Ligands Modulation of Nerve Growth Factor Bioactivity: A Structural Biology Overview

**DOI:** 10.3390/cells10123462

**Published:** 2021-12-08

**Authors:** Francesca Paoletti, Doriano Lamba

**Affiliations:** 1 Laboratory for Molecular Structural Dynamics, Theory Department, National Institute of Chemistry, SI-1001 Ljubljana, Slovenia; 2 Institute of Crystallography—C.N.R.—Trieste Outstation, Area Science Park—Basovizza, I-34149 Trieste, Italy; doriano.lamba@ic.cnr.it; 3 Interuniversity Consortium “Biostructures and Biosystems National Institute”, I-00136 Roma, Italy

**Keywords:** nerve growth factor, small endogenous ligands, bioactivity modulation, lysophospholipids, ATP, zinc

## Abstract

Experiments with cell cultures and animal models have provided solid support for the assumption that Nerve Growth Factor (NGF) plays a key role in the regulation of neuronal cell survival and death. Recently, endogenous ligands have been proposed as physiological modulators of NGF biological activity as part of this regulatory cascade. However, the structural and mechanistic determinants for NGF bioactivity remain to be elucidated. We recently unveiled, by an integrated structural biology approach, the ATP binding sites of NGF and investigated the effects on TrkA and p75^NTR^ receptors binding. These results pinpoint ATP as a genuine endogenous modulator of NGF signaling, paving the way to the characterization of not-yet-identified chemical diverse endogenous biological active small molecules as novel modulators of NGF. The present review aims at providing an overview of the currently available 3D structures of NGF in complex with different small endogenous ligands, featuring the molecular footprints of the small molecules binding. This knowledge is essential for further understanding the functional role of small endogenous ligands in the modulation of neurotrophins signaling in physiological and pathological conditions and for better exploiting the therapeutic potentialities of NGF.

## 1. Introduction

The nerve growth factor (NGF) was the first identified and the structurally and functionally best characterized member of the neurotrophin (NT) family [1]. It is involved in maintenance and growth of different neuronal populations and exerts its activity through TrkA and p75^NTR^ receptors [2]. NGF is produced as a precursor, proNGF, which is secreted by many tissues and is the predominant form of NGF in the central nervous system (CNS). The homeostasis between mature neurotrophin NGF and its precursor proNGF is thought to be crucial in physiology and in pathological states [3]. Besides its role in the nervous system, NGF also takes part in the activation of the immune and endocrine system and is involved in the pain- signaling pathways [4].

Recently, it became clear that the molecular bioactivity of NGF is associated to other actors, whose role in NGF signaling has been often overlooked. These are small endogenous biomolecules (nucleotides, lipids and carbohydrates) involved in many intracellular pathways [5,6,7,8,9]. It thus remains crucial to elucidate the molecular and chemical topology of the partners involved in neurotrophins signaling. To this aim, besides the fundamental cellular biology insights, a detailed high-resolution analysis of the binding modes of protein–small ligand complexes from 3D structure data is essential for understanding the selective ligand recognition by proteins. 

This review aims to provide an up-to-date analysis of the available 3D structural data on the binding of NGF to small endogenous ligands, with the further intention of identifying common features that are likely to contribute to the binding to molecular surfaces as well as to specific binding pockets, which might be exploited by further biological and pharmacological studies. It is out of the scope of this review the description of the binding of non-endogenous agonists/antagonists small molecules to NGF.

At first, a general overview of the biological data available for the interaction of NGF with each of the reported different small endogenous ligands is given. Then, a detailed description of all the available PDB-deposited structures (Table 1 and Supplementary Figure S1) for NGF bound to each of the reported ligands will be carried out. The review will be concluded by a general discussion with the significance of these structures in the context of the functional implication for NGF bioactivity.

## 2. NGF and Lipid Molecules

### 2.1. Biological Context

**Figure 1:**
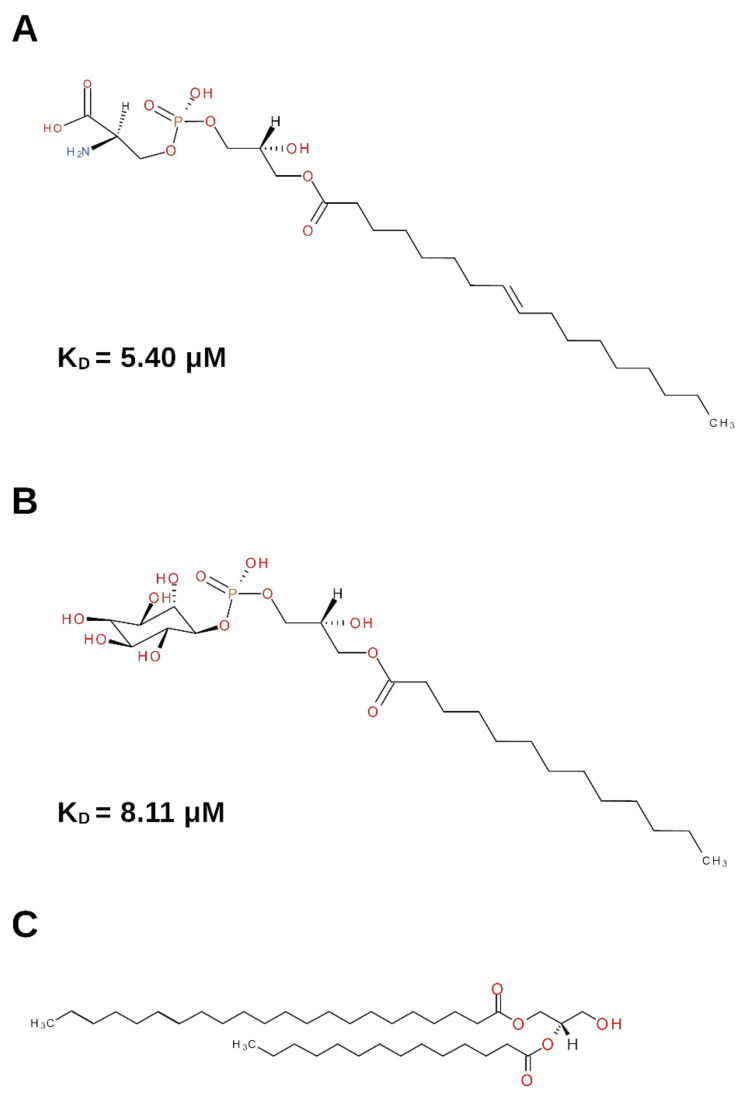
Schematic representation of the lysophospholipids involved in NGF binding. (**A**) Lysophosphatidylserine (Lyso-PS). (**B**) Lysophosphatidylinositol (Lyso-PI). (**C**) Diacylglycerol (DG). The K_D_ value of the binding of the lysophospholipids to NGF, when available, is reported.

Lipids in the nervous system play many important functions. Among them, their structural role in biological membranes, their participation as bioactive messengers involved in cell signaling, and their contribution to energy supply are very relevant. In particular, lysophospholipids (Figure 1) are emerging as endogenous ligands that affect various functions such as cell growth, differentiation and motility, in a number of cell types, including nervous cells [12,13].

Lysophosphatidylserine (Lyso-PS) has earned an increasing interest in the literature in recent years [14]. It has become clear that Lyso-PS is present in the CNS and in the immune system and its receptors have been characterized [15]. Further studies have shown that Lyso-PS is showing immunomodulatory functions, and several studies have proven an important link between Lyso-PS and neurodegenerative as well as autoimmune diseases [15]. 

Given the important role of NGF and the other neurotrophins in both the CNS and the immune system, it is plausible to hypothesize that a direct interaction between Lyso-PS and NGF might be of important functional significance. It has been shown that Lyso-PS is involved in histamine degranulation in mast cells [15]. Different sets of experiments have proven that there is a powerful synergism between Lyso-PS and NGF in mast cells, activating histamine secretion at low Lyso-PS concentrations [16,17] and in the presence of extracellular Ca^2+^ [18] and phospholipase D [19]. This mechanism was shown to be active through TrkA receptor. Other members of the neurotrophin family do not show this same NGF activity in mast cells [20]. The mast cell effect of Lyso-PS and NGF has been also shown to involve activate platelets, thus suggesting an important role of NGF in chronic inflammation and wound healing [21]. Also, Lyso-PS has been proven to enhance NGF-induced neurite outgrowth in PC12 cells [22], thus confirming that the Lyso-PS-NGF synergy is effective also in neuronal cell types. This effect was suggested to be of significance in neurons in the case of cell damage or inflammation [22].

Not only Lyso-PS, but also lysophosphatidylinositol (Lyso-PI) has been identified as a strong binder for NGF [7], and the X-ray crystal structure of the complex was solved (PDB ID: 4XPJ [7]). An increasing literature evidence points towards a significant role for Lyso-PI in many cellular contexts, including the physiopathology of neuronal cell types [12,23]. To the best of our knowledge, no functional/biological studies have been reported so far elucidating the role of the Lyso–PI/NGF interaction. Lyso-PI was found to induce neurite retraction through its receptor GPR55 in NGF-differentiated PC12 cells [24]. Given that Lyso-PI is emerging as an important bioactive lipid that can function as a cell-growth modulator, it might be speculated that the Lyso-PI/NGF might be of functional importance [7].

It was also reported that lysophosphatidylcholine specifically enhances NGF-induced signals in PC12 cells, affecting the TrkA signaling pathway [25]. It remains of interest to unravel the dynamic behavior as well the functional consequences upon receptor binding.

### 2.2. Structural Data

The X-ray crystal structures of mouse NGF in complex with Lyso-PS [6] (PDB ID: 4EAX), and Lyso-PI [7] (PDB ID: 4XPJ) were reported. In addition, the X-ray crystal structure of NGF isolated and purified from Chinese cobra venom (cNGF) unveiled the incorporation of the diacylglycerol (DG) (2S)-1-hydroxy-3-(tetradecanoyloxy)propan-2-yl docosanoate into the structure (PDB ID: 4EC7) [6].

#### 2.2.1. (2.S)-1-Hydroxy-3-(tetradecanoyloxy)propan-2-yl docosanoate—Diacylglycerol (DG) 

**Figure 2:**
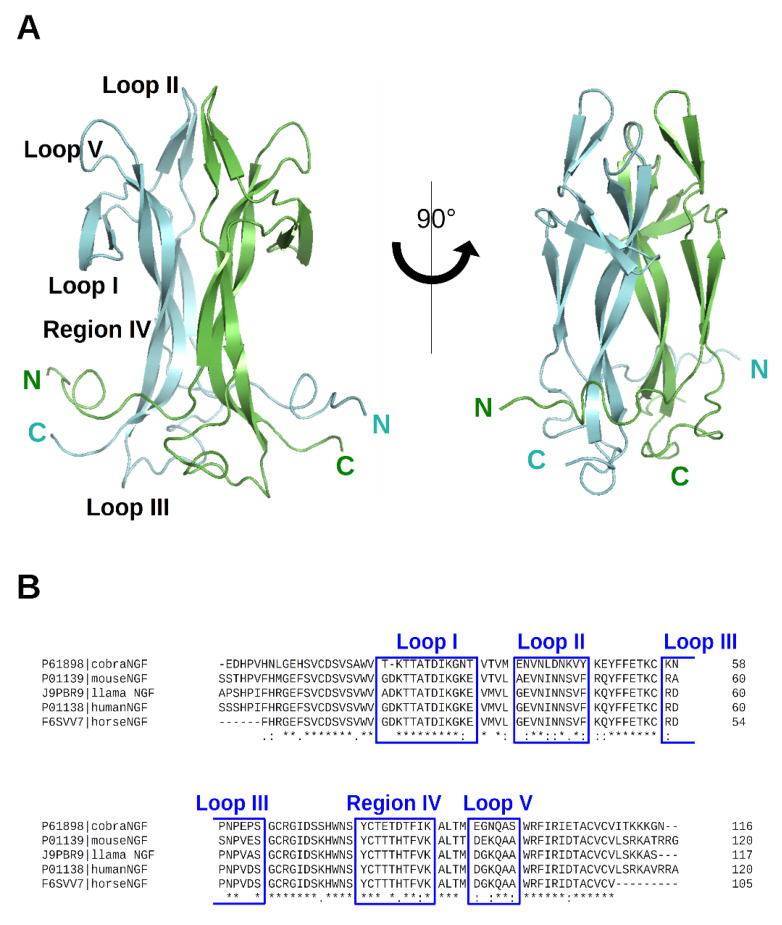
(**A**) 3D structure of NGF (PDB ID: 6YW8 [5]). The two protomers are colored in cyan and green, respectively. N- and C-termini are labeled. Loops have been labelled according to Ibanez [27]. Two different protein orientations are shown. Figures were produced using PyMOL [28]. (**B**) Sequence alignment of the NGF of the different species for which a PDB-deposited 3D structure is available. The alignment was performed with ClustalW webserver [29]. UniProt entry numbers are indicated. Loops have been labelled according to Ibanez [27].

The crystal structure of cNGF was determined at 2.60 Å resolution (PDB ID: 4EC7) [6]. It adopts a very close secondary structural scaffold if compared with the previously reported apo structure of mouse NGF (mNGF) (PDB IDs: 1BTG [10]; 1BET [26]). The protomer architecture of cNGF comprises four antiparallel twisted beta-strands that are connected at one end by a reversed turn (Loop III) and three beta-hairpin loops (Loop I, Loop II, and Loop V) at the other end (Figure 2).

**Figure 3:**
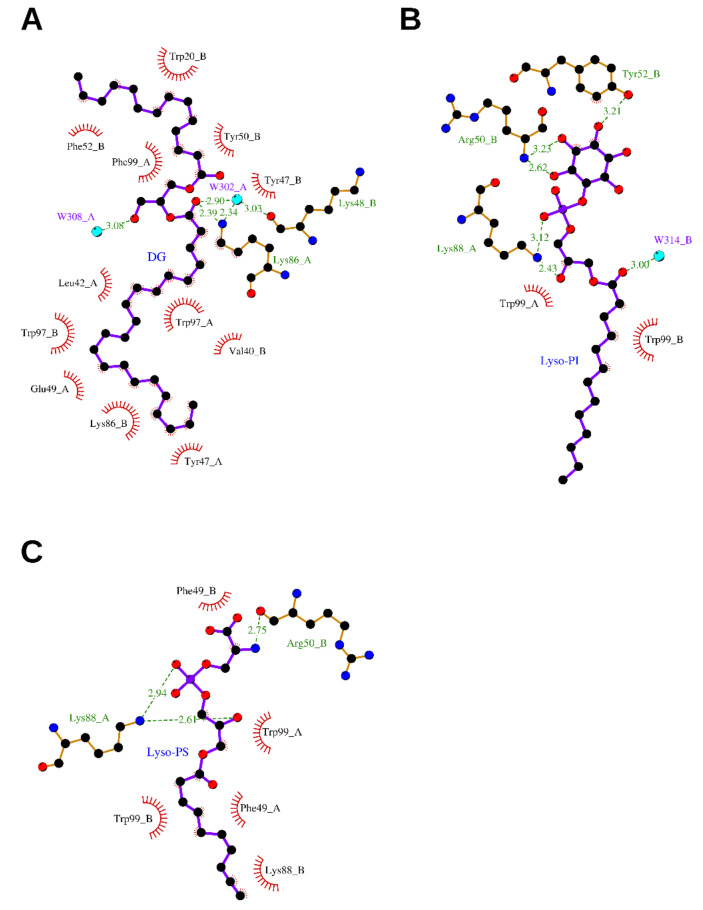
Binding sites of mNGF/hNGF/cNGF complexed with DG (**A**), Lyso-PI (**B**), Lyso-PS (**C**), respectively. The ligands and the protein side chains are shown in ball-and-stick representation, with the ligand bonds colored in purple. Hydrogen bonds are shown as green dotted lines, while the spoked arcs represent protein residues making nonbonded (hydrophobic) contacts with the ligand. The 2D ligand–protein interaction diagrams have been produced with LigPlot+ [30].

An elongated tube-shaped electron density was identified to span the molecular cNGF homodimer. A Mass Spectrometry (MS) analysis of the organic compounds extracted from purified cNGF pointed to a molecular entity of 620 Da [6]. The overall shape and the chemical environment of the ligand electron density let to hypothesize that the bound molecule was a 2-tailed fatty acid ester (no apparent electron density could be ascribed to the presence of a phosphate, sulfate or sugar moiety). However, attempts identifying this lipid by biophysical methods such as Nuclear Magnetic Resonance (NMR) were hampered by the rather low abundance of cNGF in the venom. Nevertheless, to gain insights into lipids binding to NGF, based on the shape of the electron density map, a putative DG molecule was tentatively modeled into the bifurcated asymmetric binding site. The molecule is anchored to cNGF *via* hydrogen-bonding interactions between its polar head and the neighboring residue Lys86_A, and by extensive hydrophobic interactions between its two alkyl tails and several hydrophobic residues. The longer tail (22 carbons) is bent in the middle to form a U shape and is buried in an inner tunnel, which is clamped by hydrophobic residues, including Tyr47_A, Tyr47_B, Tyr50_A, Trp97_A, Trp97_B, and Phe99_B. The shorter tail (14 carbons) accommodates in a surface groove at the dimeric ridge, which is lined with hydrophobic residues, including Trp20_B, Ile29_A, Phe52_B, Phe84_A, and Phe99_A. Lys86_A and Lys86_B adopt different conformations, which may likely be attributed to the asymmetry endowed by the lipid-bound pocket (Figure 3A and Supplementary Figure S1).

#### 2.2.2. (2.S)-2-Amino-3-[hydroxy-[(2R)-2-hydroxy-3-octadecanoyloxypropoxy]phosphoryl] oxypropanoic acid—Lysophosphatidylserine (Lyso-PS)

Intriguingly, the crystal structure of mNGF purified from mouse submaxillary glands lacks lipid-binding ability or is simply in a lipid-free state (PDB IDs: 1BTG [10], 1BET [26]). Despite the fact that the crystal forms differ, this finding is consistent with the very first crystallographic study on mNGF. To explore further the chemical and structural determinants of lipid recognition mechanism by NGF, attempts to purify and crystallize mNGF in complex with Lyso-PS, Lysophosphatidic acid, or phosphatidylinositol-4,5-bisphosphate, respectively, successfully resulted in achieving high-quality crystals of mNGF only in complex with Lyso-PS, the structure of which has been determined at 2.30 Å resolution (PDB ID: 4EAX) [6]. 

A continuous finger-shape electron density map was clearly detected, which indicated that the Lyso-PS molecule was bound. In contrast to the crystal of cNGF, in which only a single-lipid orientation could be accommodated, the featureless electron density map in mNGF resolved in modelling two identical Lyso-PS molecules with opposite orientations. 

The polar head and C1–C8 of the Lyso-PS alkyl chain can be readily modeled according to the electron density map. On the contrary, region C9–C18, located in the solvent region, resulted instead to be not traceable due to extremely poor quality of the electron density map. The conformation of Lyso-PS differs from that of the longer tail of the DG lipid in cNGF; however, Trp99_A/B and Phe49_A/B (corresponding to Phe97_A/B and Tyr47_A/B in cNGF, respectively—Figure 2B) clamp both molecules. The C5–C8 portion of the Lyso-PS molecule adopts a nearly fully extended conformation, and it is located at an average of 2–3 Å above the U-shaped longer tail of the DG in cNGF, which suggests that the pocket is able to accommodate lipid tails of different lengths. The head group of Lyso-PS is in a nearly identical location to that of the lipid molecule in cNGF, with both the OH of the head glycerol group and the phosphate group interacting with Lys88 of mNGF. The serine moiety of Lyso-PS is exposed to the solvent region and interacts with Asn45 of the Loop II from a neighboring dimer. However, the interaction between Lyso-PS and Asn45 is likely the result of crystal packing contact, which is in line with its failure to induce tetramerization of NGF in solution. The serine moiety of Lyso-PS may likely act as an anchor point for other cellular components (Figure 3C). 

An overall structural comparison of the Lyso-PS complexed structure with native mNGF revealed marked differences that were principally located at hairpin Loop II, which is formed by residues 41–49 (Figure 2). In the lipid-free mNGF structure, the two Loops II are bent toward the NGF dimer interface, whereas in the complexed structure, the Loops II point outwards from the NGF dimer interface (Supplementary Figure S1A). In the cNGF–DG complex (PDB ID: 4EC7) [6], the conformation of the Loops II lies between these two states (Supplementary Figure S1C). 

A notable conformational rearrangement of the Lys88 residue of mNGF was observed upon binding of Lyso-PS (Figure 3C), suggesting that Lys88 may play an essential role in the recognition of the lipid head groups. Displacement of the lipid-contacting residues Trp99_A/B and Phe49_A/B of mNGF was also observed upon binding of Lyso-PS (Figure 3C). Due to this difference in Loop II conformation, the pocket results to be a solvent-inaccessible “tunnel” in cNGF-DG (Figure 3A, Supplementary Figure S1), whereas an exposed and wide “groove” in mNGF: Lyso-PS (Figure 3B, Supplementary Figure S1).

#### 2.2.3. 1-Tridecanoyl-2-hydroxy-sn-glycero-3-phospho-(1′-myo-inositol)—Lysophosphatidylinositol (Lyso-PI)

To expand further the understanding of the structural basis for specific lipid recognition by NGF, the crystal structure of mNGF complexed with Lyso-PI was determined at 2.60 Å resolution (PDB ID: 4XPJ) [7]. At the dimeric interface, one Lyso-PI molecule, with a fully modelled alkyl tail (C1–C13), is clamped by the mNGF dimer *via* both hydrogen-bonding interactions between its head group and polar residues and extensive hydrophobic interactions between its alkyl tail and the pocket hydrophobic residues. The mNGF–Lyso-PI complex structure differs with respect to the native mNGF structure, namely, in the conformation of the hairpin Loop II formed by residues 41–49, especially Asn45 that points straight up to form a cavity, which is likely induced by lipid binding and similar to that found in the mNGF–Lyso-PS complex structure. The overall Lyso-PI conformation differs from that of the lipid in cNGF but is similar to that of Lyso-PS in the mNGF–Lyso-PS complex structure. The head glycerol groups of Lyso-PI and Lyso-PS are in a nearly identical position (Supplementary Figure S1), while the inositol moiety of Lyso-PI is exposed to the solvent and the interaction patterns are different (Figure 3B).

In addition to Lys88, which interacts with the head glycerol group and the phosphate group of Lyso-PI, as in the mNGF-Lyso-PS structure, two additional residues, Tyr52 and Arg50, were found to assist in lipid binding by forming hydrogen bonds to the inositol moiety of the Lyso-PI molecule. The phenolic hydroxyl of Tyr52 is hydrogen-bonded to the 4-OH group of the Lyso-PI inositol moiety, and the main chain nitrogen of Arg50 interacts with the 3-OH group of the Lyso-PI inositol moiety.

## 3. NGF and Molecules Carrying Phosphate Groups

### 3.1. Biological Context

It was reported recently that ATP could bind proteins lacking a *bona fide* ATP-binding domain. This is also true for ATP binding to NGF. The binding was proven by MS data and the formed complex was identified as to be protective towards hippocampal neural cells from death [31,32,33,34]. Similar observations were reported for the other member of the NT family, Brain-Derived Neurotrophic Factor (BDNF) [35], as well as for other growth factors (Fibroblast Growth Factor (FGF) [36], Vascular Endothelial Growth Factor (VEGF) [37]). Despite these data, a full understanding of the physiological role of the extracellular ATP–NGF interrelationship remains, however, to be elucidated [32,33]. 

The extracellular ATP levels in the cells of the nervous system are finely tuned [38,39]. In physiological conditions, low extracellular ATP levels are reported, while increased extracellular ATP concentrations up to mM have been described to be responsible for cell damage or inflammation. In fact, a variation of extracellular ATP levels in the brain has been detected in physiological or pathological conditions and related to the activation of specific subclasses of P2 receptors [38,39]. The finely modulated purinergic signaling suggests that its alteration might affect several human diseases, including neurodegenerative disorders as well as inflammatory dysfunction and tumors [39]. A link between the ATP purinergic signaling system and the neurotrophins signaling has been described in a set of crucial cellular studies in different disease states [40,41]. The interaction of both ATP and NGF with their receptors has been reported to be ascribed to their relative extracellular levels. An unbalance in the extracellular levels of both ATP and NGF would thus be responsible for the cellular response to cell damage. At the same time, an active role of ATP into PC12 cells activation was reported, in synergy with NGF [41]. 

Recently, a study has been reported describing a thorough structural/biophysical study to characterize the interaction of ATP with recombinant human NGF (rhNGF). This study describes the molecular determinants of the interactions between ATP and rhNGF and allows to unveil the previously unknown binding sites on the molecular surface and the ligand’s orientation [5]. According to Isothermal Titration Calorimetry (ITC) data, ATP binds rhNGF with a low affinity (mM range), as confirmed by Surface Plasmon Resonance (SPR) and Saturation Transfer Difference NMR (STD-NMR). This affinity collocates the binding among the transient interactions with weak affinities, named “quinary interactions”, which are gaining importance in the understanding of the cell regulation [42,43,44] and its dynamic processes needed for cell survival. Recently, several studies reported on significant roles for ATP at millimolar concentration [45,46,47]. The functional role of the ATP-rhNGF interaction in the context of TrkA and p75^NTR^ receptors was studied by SPR. Also, the investigation of the functional role of divalent ions (Mg^2+^ or Zn^2+^) in conjunction with ATP allowed to conclude that the relative stoichiometry of ATP-ion-rhNGF is a key factor for the regulation of the binding effect [5]. The structural/computation study pinpoints ATP as a genuine endogenous modulator of NGF signaling in vivo, in health and disease conditions. It is tempting to speculate that ATP might be involved in the amplification of NGF signaling in physiological conditions through TrkA receptor, as reported [34]. On the contrary, neurodegeneration or cellular stress has been associated with massive release of extracellular ATP [39] and a concomitant reduction of TrkA receptor signaling [48]. Furthermore, millimolar ATP concentrations can activate P2X receptors in trauma conditions and trigger the prevention of a large inflammatory response [38,49]. A direct ATP-hNGF binding might thus assume the function of a molecular switch between NTs versus purinergic receptors systems to adapt to the cellular needs.

It is interesting to mention that the NGF has also been found in llama seminal plasma as the ovulation-inducing factor (OIF). Once the X-ray crystal structure of the protein was solved (PDB ID: 4EFV [8]), it revealed the presence of a phosphate group in close vicinity to the ATP binding site reported by Paoletti et al. [5]. This further corroborates the importance of phosphate ion carrying ligands to NGF.

### 3.2. Structural Data

#### 3.2.1. Adenosine-5′-Triphosphate (ATP)

Hunting for the structural and functional characterization of new endogenous modulators of hNGF biological activity, the binding properties of ATP to rhNGF were investigated. 

Fourier Transform–Infrared Spectroscopy (FT–IR) measurements ruled out changes in the secondary structure of rhNGF due to the interaction with ATP. SPR, ITC and 1D ^1^H STD-NMR pinpoint to low affinity (mM range) ATP-rhNGF binding. ITC titration data returned a K_D_ of 1.38 mM, underlining transient interactions with weak affinities [5].

Differential Scanning Fluorimetry (DSF) explored the effects of different divalent cations on the binding interactions between ATP and rhNGF. A major effect of ATP on rhNGF thermal stability occurred in the presence of Zn^2+^. SPR analysis confirmed that Zn^2+^ markedly affected ATP binding to rhNGF. 1D ^1^H STD-NMR experiments were used to determine the binding epitope of ATP when bound to rhNGF in the presence of either Mg^2+^ or Zn^2+^ ions. In either case, the strongest interaction was observed for H2 proton on the adenine and H4′ proton on the sugar moieties of ATP, respectively.

**Figure 4:**
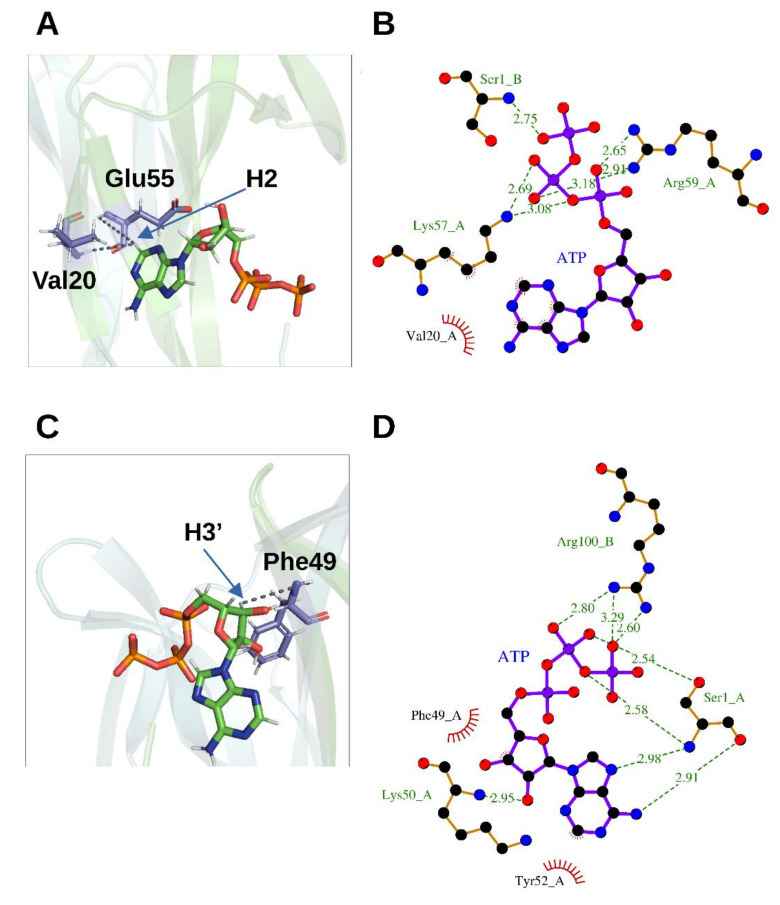
Binding orientation of ATP on rhNGF. (**A**,**C**) Representative poses from MD analysis for Site 1 (**A**) and Site 2 (**B**). Cyan and green transparent cartoon: rhNGF protomers; ATP is represented as colored by element (C—green; N—blue; O—red; H—white; P—orange). Residues V20, E55 and F49 showing new NOEs upon ATP binding are labelled and colored by element (C—violet; N—blue; O—red; H—white). Black broken lines represent the distances between HN protons of rhNGF and ATP protons (indicated by a blue arrow). Figures produced using PyMOL [28]. (**B**,**D**)—Binding sites of rhNGF complexed with ATP for Site 1 (**B**) and Site 2 (**D**), respectively. The ligands and protein side chains are shown in ball-and-stick representation, with the ligand bonds colored in purple. Hydrogen bonds are shown as green dotted lines, while the spoked arcs represent protein residues making nonbonded (hydrophobic) contacts with the ligand. The 2D ligand–protein interaction diagrams have been produced with LigPlot+ [30].

2D ^1^H-^15^N HSQC spectra following a titration with increasing amounts of ATP allowed characterizing the rhNGF residues engaged in the interactions with ATP. The analysis of the combined ^1^H/^15^N Chemical Shift Perturbation (CSP) showed residues that are likely involved directly in ATP binding, *i.e.*, Trp21, Ile31, Asn45, Phe49, Thr56, Leu90, Ala97 and Ile 104. 3D ^15^N-NOESY-HSQC spectrum collected at ATP-rhNGF titration endpoint unveiled ATP binding to two previously unknown sites on each rhNGF protomer, with a 4:2 (ATP:rhNGF) stoichiometry. The site encompassing residues Val20, Trp21, Val22 and Glu55 has been named as “Site 1” (Figure 4A,C) and that defined around Phe49, namely, residues encompassing residues Thr29 and Lys34 (Loop I) and Ser47 and Val48 (Loop II) as “Site 2” (Figure 4B,D). The potential energy differences (only the enthalpic term has been considered) were calculated, with respect to the unbound state, for the ATP-rhNGF interactions: these values are −14.6 ± 1.2 kcal/mol for “Site 1” and −2.34 ± 1.3 kcal/mol for “Site 2” [5].

An earlier study [33] based on MS and mutagenesis studies reported the involvement of the C-terminus of rhNGF in ATP binding. Our data instead clearly show no involvement of this region in rhNGF binding.

#### 3.2.2. Phosphate Ion

**Figure 5:**
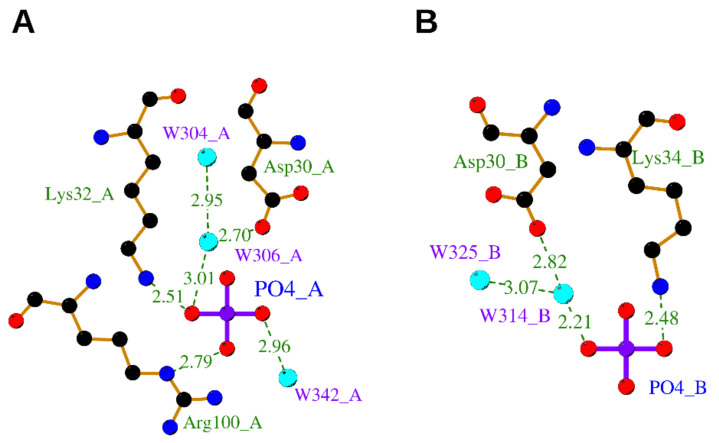
Binding sites of llama NGF complexed with phosphate ion. The binding of phosphate ion with protomer A and B is shown in panels (**A**,**B**), respectively. The ligands and protein side chains are shown in ball-and-stick representation, with the ligand bonds colored in purple. Hydrogen bonds are shown as green dotted lines. The 2D ligand–protein interaction diagrams have been produced with LigPlot+ [30].

An OIF in the seminal plasma of llama has been isolated and identified as nerve growth factor [8]. Its crystal structure determined at 2.32 Å resolution (PDB ID: 4EFV) [8] unveiled that a phosphate ion is bound on each OIF protomer. Remarkably, the phosphate is engaged in hydrogen bonds to the highly conserved residues Asp30 and Lys32 both belonging to Loop I and Arg100 belonging to Loop V, closely resembling the “Site 2” terminal phosphate moiety of ATP in hNGF (Figure 5).

## 4. NGF and Zinc

### 4.1. Biological Context

The involvement of divalent cations in the binding to NGF is of great interest. It is well known that Zn^2+^ is involved in the physiological metabolism of different cell types, as well as it is known that a dysfunction in Zn^2+^ metabolism plays a role in many pathological states [50], including neurodegenerative diseases [51] and brain ischemia [52]. Indeed, a cross-talk between metal ions homeostasis and neurotrophins in the explanation of the Alzheimer’s Disease (AD) molecular pathways has been proposed [53]. 

Several studies report on the binding of Zn^2+^ and other divalent cations to NGF [54,55]. Over the years, several groups reported on the effect of Zn^2+^ on NGF structure and activity [9,55,56]. Initially, zinc was related to the structure and function of NGF since the early studies [57,58]. However, in this case, zinc is related to the γ subunit of the 7S NGF complex in mice salivary glands and it is actively involved in its bioactivity and autocatalytic activation [57,59,60]. The subsequent X-ray structure of the 7S NGF complex revealed that, in fact, Zn^2+^ is bound between the α1 and γ1 subunits (PDB ID: 1SGF [61]). Interestingly, in an early study on the role of zinc on NGF function, it has been proposed that Zn^2+^ might be related to His8 on the β subunit of the 7S NGF, which is, in fact, the active neurotrophic subunit [62]. It has been subsequently proposed by molecular modeling studies that Zn^2+^ binding to NGF involves the N-terminal histidines (His4 and His8), as well as the stem residues (His84 and Asp105) [54]. Changes in NGF conformation in the presence of Zn^2+^ were reported [54,56]. The effects on secondary structure changes induced by this binding are, overall, well accepted, although the effect on the secondary structure appears to be dependent on the stoichiometric ratios between Zn^2+^ and NGF, as well as on the used cation concentrations [9,55]. This is not surprising, considering that it is well known that zinc homeostasis needs to be tightly regulated, specifically in neuronal populations [50]. The effects of Zn^2+^ on NGF are not only limited to a change in its secondary structure. These effects influence the biological NGF activity [54,63]. Indeed, Zn^2+^ was established to inhibit both TrkA- and p75^NTR^-mediated effects of NGF [54,63,64]. 

One of the first deposited PDB mouse NGF structures (PDB ID: 1BTG) [10] reported the presence of a Zn^2+^ linking two NGF protomers of different dimers. Interestingly, the Zn^2+^ was also found in the recently PDB-deposited structure of horse NGF (PDB ID: 6XUO—no related publication available).

### 4.2. Structural Data

The structure of the bis-desocta_1-8_ form of murine βNGF has been determined in two different crystal forms using X-ray methods. The two crystal forms, with space groups P2_1_2_1_2_1_ and C2, were grown from a precipitant cocktail containing zinc acetate concentrations of 1 mM and 100 mM, respectively. 

A zinc-binding site in the “waist” of the molecule near His84 and Asp105 has been identified. 

A total of three Zn^2+^ bind with different stoichiometry to each of the three protomers in the C2 crystal forms (PDB ID: 1BTG) [10]. Both protomers in the P2_1_2_1_2_1_ crystal form instead have one ion bound per protomer. In both cases, the metal ions are held less tightly than in the C2 crystal form (Table 2). In each crystal forms, Zn^2+^ are bound at site away from a crystal contact, suggesting that the protein has a genuine affinity for zinc.

One can speculate that NGF might interact with one of its receptors via the His84/Asp105/Zn^2+^ complex. This is consistent with the evidence that residue 84 is crucial in TrkA receptor binding [65]. Studies of the binding of NGF to p75^NTR^ receptor showed that divalent ions (Zn^2+^ and Cu^2+^) are able to antagonize p75^NTR^-driven apoptosis in chick neural retina and are able to block NGF binding to p75^NTR^ receptor and consequently attenuate its pro-apoptotic signaling cascade in chick embryonic cell cultures [64]

In the recently determined crystal structure of horse NGF (PDB ID: 6XUO) [11], two Zn^2+^ have been identified to bind each protomer. Crystals with space group I2_1_3 were grown from a precipitant cocktail containing a zinc acetate concentration of 110 mM. Namely, Zn^2+^(1) is coordinated by Asp24 and by His 84 and Asp105 (both belonging to a symmetry related protomer), in analogy to what is being observed in mNGF crystal structure. Zn^2+^(2) is surrounded instead by the carboxyl moieties of three different Glu35 residues, each of them belonging to three different symmetry-related protomers. Zn^2+^ likely mediates packing contacts in the horse crystal structure (Supplementary Figure S1). 

## 5. Conclusions and Future Perspectives

The present 3D structural overview provides detailed and solid evidence pinpointing the important role of small endogenous ligands in the modulation of the biology of NGF, as well as highlighting both common and different molecular determinants in the binding mode of the endogenous ligands. Such information is important to get a complete picture of the regulation of the bioactivity of NGF in health and disease states. 

**Figure 6:**
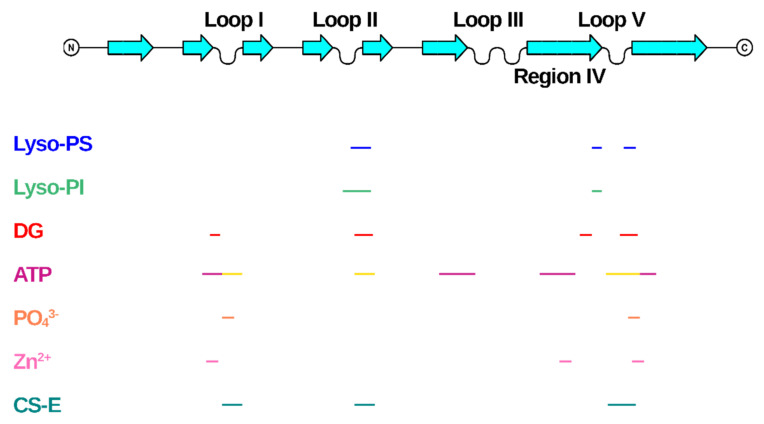
Mapping of the binding regions of the endogenous small ligands on NGF structure. Schematic drawing of NGF primary structure with indication of secondary structure elements and loops names according to Ibanez [27]. Figure generated using Top Draw sketchpad [66]. The stretches of residues involved in the binding to the different small endogenous ligands are indicated by different color codes. Lyso-PS: blue; Lyso-PI: green; DG: red; ATP: site 1—magenta, Site 2—yellow; PO_4_^3−^: orange; Zn^2+^: pink; CS-E: teal.

In all the reported cases, it clearly emerges that the binding of the endogenous ligands induces a change in the NGF structure, thus demonstrating an unexpected structural plasticity of a quite compact structure. All the ligands bind in regions (pockets or surfaces) of NGF far away from the cystine-knot moiety, thus indicating that the latter represents a nucleation motif, the topology of which is essential for the folding of a biologically functional shape (Figure 6).

The largest molecules considered in our survey are lipid molecules. Interestingly, these molecules involve an opening of the Loop II region in NGF able to create a cavity to accommodate the lipids (Supplementary Figure S1). This is the largest structural rearrangement involving NGF binding. It has been suggested by molecular modelling that the lysophospholipids binding to NGF might interfere with the NGF binding to p75^NTR^ receptor more than it would do to the TrkA receptor interaction [6]. It remains to be proven experimentally whether this is the case and what would be the physiological implications.

Interestingly, the Loop II involved in the lysophospholipids binding is also partly involved in the binding to phosphate ion and ATP (Figure 6). This observation thus points to this region of NGF as a functionally flexible region, and possibly an interaction hotspot with a regulatory role on the downstream NGF signaling. 

It is tempting to hypothesize a binding mechanism in which a pre-organized set of NGF side chains (Lys and Arg) assists at least in part in the desolvation of the phosphate moiety of the ligands into well-defined sites suitable for molecular recognition by elec-trostatic interactions.

On the contrary, the so-called Site 1 identified in the ATP binding [5] closely resembles the NGF structure region involved in the binding of Zn^2+^ atoms in different PDB-deposited structures (Figure 6). However, the structural role of Zn^2+^ in the modulation of NGF bioactivity is difficult to be ascertained, only based on the binding sites being observed in the PDB-deposited structures. Indeed, the Zn^2+^ binding sites on NGF surface differ in the mNGF and horse NGF structures, respectively (PDB IDs: 1BTG, 6XUO) [10,11].

The direct interaction of NGF with other small endogenous ligands has been reported, although no 3D experimentally determined structural details are available. The interactions of NGF with glycosaminoglycans were unraveled. Chondroitin sulfate proteoglycans (CSPGs) were found to interact with NGF [67], which has shown preferential binding to CS-E [68]. NGF-TrkA mediated phosphorylation of Akt by CS-E has been reported [68]. A detailed molecular knowledge of this CS-NGF/proNGF-TrkA molecular interaction is therefore of interest [69]. So far, only computational docking studies coupled to carbohydrate microarray investigated the molecular interactions with glycosaminoglycans, particularly CS-E [67]. Although no 3D structural data are available, it is interesting that the proposed binding site for CS-E on NGF largely overlaps to the identified Site 2 in the ATP-NGF binding studies (Figure 6), thus suggesting this site as being a likely promiscuous site for different endogenous ligands.

The different biological nature of the different small endogenous ligands and the partial overlap of the NGF regions involved in their binding allow to speculate that the modulation of NGF bioactivity might be limited to conformational flexible regions of NGF structures, namely Loops I, II and V. These regions represent therefore true “hotspots” for the small molecules binding (Figure 6). 

Given the different physiological functions, as well as their diverse distribution in different cell types, and the alterations in their binding strength to NGF, it is tempting to speculate that the small endogenous ligands described in this review might play a significant role in the modulation of NGF bioactivity. Their binding to NGF might be related not only to the physiology of NGF, but also to its involvement in the pathogenesis of a large spectrum of neuronal diseases. Given the important downstream signaling of NGF inducing important intracellular outcomes, it seems to be plausible that a regulation of the binding to its receptors might be critical, to adapt the intracellular response to the specific need in a specific timepoint and place. The identification of a possibly promiscuous binding site on NGF (Figure 6) where different small endogenous ligands could bind in different cellular contexts seems to be a way to quickly respond to an external insult or to trigger changes in the signaling pathway in response to different extracellular stimuli.

One open question to be addressed is the role that small endogenous ligands may play in modulating the bioactivity of the NGF precursor, proNGF, as well as the regulation of the proNGF/NGF ratio. Given the different biological role of these two forms of the neurotrophin, it would be of great interest to correlate their different biological outcomes to differences in their modulation by endogenous factors. The intrinsically unstructured nature of proNGF, despite some successful although partial attempts to unveil its detailed 3D structure by a multidisciplinary structural biology approach [70,71,72,73], still poses insurmountable challenge to address this key and biologically relevant issue.

Despite NGF discovery dates to seventy years ago, its biology and modulatory roles in health and disease are far from being completely unraveled. The role of endogenous ligands in the modulation of NGF and the biological activities of other neurotrophins still represents a new arena of research that has remained unexplored with key questions still awaiting answers.

## Figures and Tables

**Table 1:** Summary of the deposited PDB structures of NGF in complex with small endogenous ligands.

Type of Ligand	NGF Species	PDB Entry	Figure	Reference for the Structure	Biological Effect of Ligand/NGF Interaction
Lyso-PS (lysophosphatidyl-serine)	Mouse	4EAX	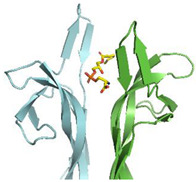	[6]	Activation of histamine secretion. Through platelet activation, role in chronic inflammation and wound healing. Enhanced NGF-induced neurite outgrowth in PC12 cells.
Lyso-PI (lysophosphatidyl-inositol)	Mouse	4XPJ	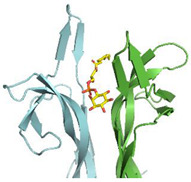	[7]	Lyso-PI induces neurite retraction through GPR55 receptor in NGF-differentiated PC12 cells.
(2S)-1-hydroxy-3-(tetradecanoyloxy) propan-2-yl docosanoate	Cobra	4EC7	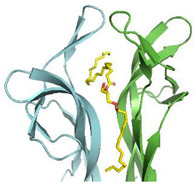	[6]	Role in histamine release.
(PO_4_)^3−^	Llama	4EFV	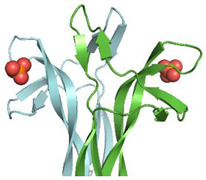	[8]	ATP binding proven to be protective towards neuronal cell death.
Zn^2+^	Mouse	1BTG	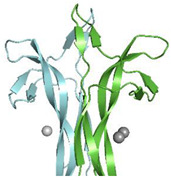	[10]	Induction of secondary structure changes on NGF. Inhibition of TrkA-and p75^NTR^-mediated effects.
Zn^2+^	Horse	6XUO	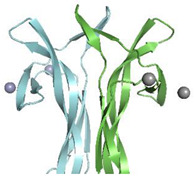	[11]	To be published.

**Table 2:** Description of the characteristics of the space groups of NGF-Zn^2+^ structures.

Crystal Form	Ion	Protein Ligand	Distance (Å)
*P*2_1_2_1_2_1_	Zn^2+^ (1)	His84 (Protomer 1)	2.4
		Asp105 (Protomer 1)	2.1
	Zn^2+^ (2)	His84 (Protomer 2)	2.3
		Asp105 (Protomer 2)	2.0
C2	Zn^2+^ (1)	His 84 (Protomer 3)	2.0
		Asp 105 (Protomer 3)	2.3
	Zn^2+^ (2)	Glu 94 (Protomer 1)	1.9
		Asp 105 (Protomer 3)	1.9
	Zn^2+^ (3)	His 84 (Protomer 2)	2.0
		Asp 105 (Protomer 2)	2.0

## Data Availability

Not applicable.

## Supplementary Materials

The following are available online at https://www.mdpi.com/article/10.3390/cells10123462/s1, Figure S1: 3D structure of the deposited PDB structures of NGF in complex with endogenous ligands.
