# Supplementary material for: Small Endogenous Ligands Modulation of Nerve Growth Factor Bioactivity: A Structural Biology Overview
Source: Cells. 2021 Dec 8;10(12):3462. doi: 10.3390/cells10123462 (PMC8700322; doi:10.3390/cells10123462)
Supplement: Supplementary file 1 [file cells-10-03462-s001.zip › cells-1473615-supplementary.pdf]

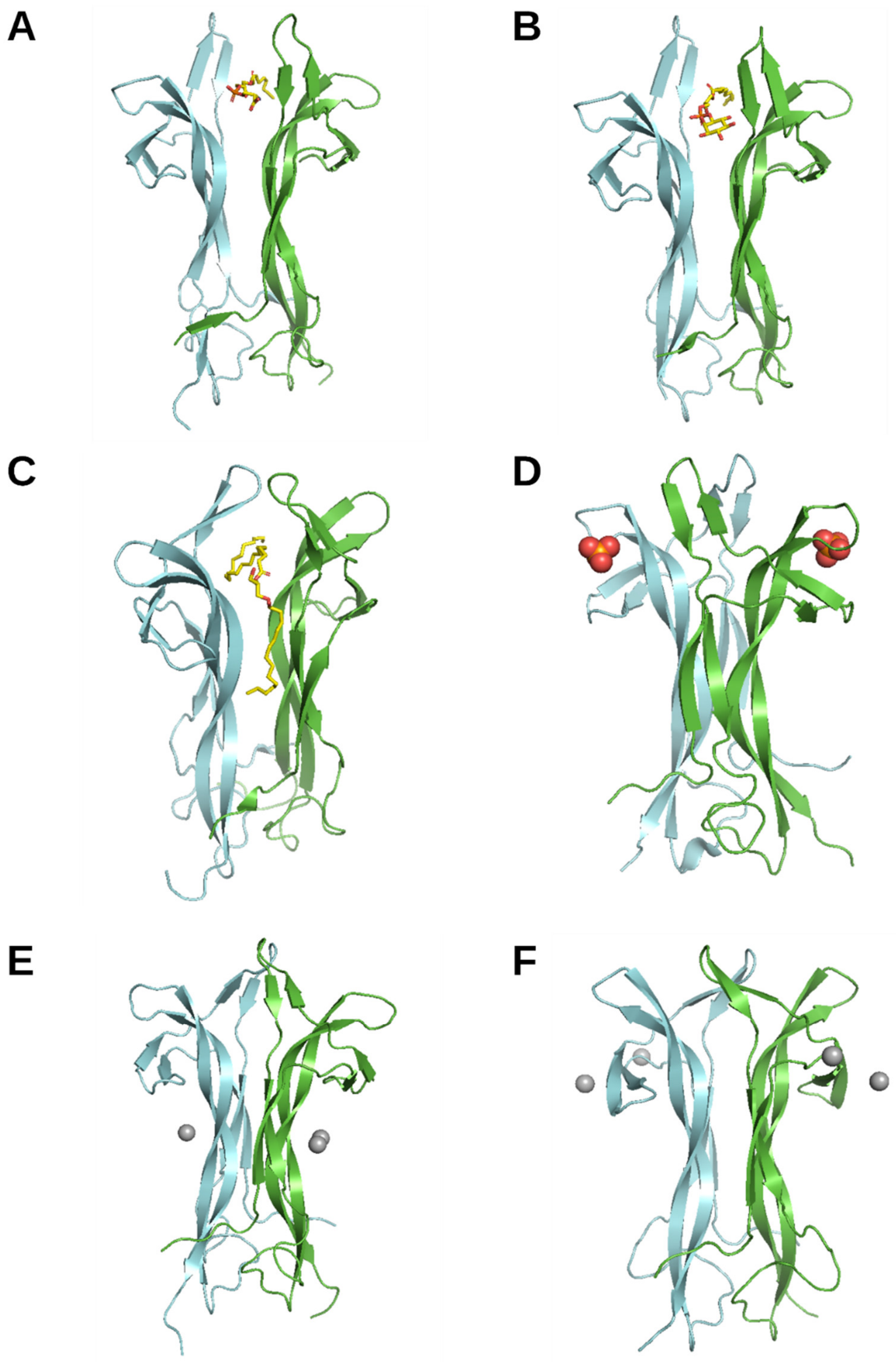

Figure S1. 3D structure of the deposited PDB structures of NGF in complex with endogenous ligands. Compare also Table 1 in main text. (**A**) mouse NGF in complex with lysophosphatidylserine (PDB ID: 4EAX) [6] (**B**) mouse NGF in complex with lysophosphatidylinositol (PDB ID: 4XPJ) [7] (**C**) cobra NGF in complex with (2S)-1-hydroxy-3-(tetradeca-noyloxy) propan-2-yl docosanoate (PDB ID: 4EC7) [6] (**D**) Llama NGF in complex with $(PO_4)^{3-}$ (PDB ID: 4EFV) [8] (**E**) Mouse NGF in complex with Zn^{2+} (PDB ID: 1BTG) [10] (**F**) Horse NGF in complex with Zn^{2+} (PDB ID: 6XUO) [11] . The two NGF protomers are colored in cyan and green, respectively. Ligands in panels (**A**–**D**) are represented as colored by element (C–yellow; N–blue; O–red; P–orange). Zinc ions in panels (**E**–**F**) are represented by grey spheres. Figures produced using PyMOL [28].
